# Formulation and Analytical Characterization of Phenprocoumon-Loaded κ-Carrageenan Hydrogels for Controlled-Release Applications

**DOI:** 10.3390/molecules31142540

**Published:** 2026-07-22

**Authors:** Iulia Gallo, Camelia Epuran, Ion Fratilescu, Raul Ștefan-Pantiș, Alexandru Pahomi, Mihaela Maria Budiul, Titus Vlase, Gabriela Vlase

**Affiliations:** 1 Research Centre for Thermal Analysis in Environmental Problems/ICAM-Advanced Environmental Research Institute, West University of Timisoara, Oituz Street 4, 300233 Timisoara, Romania; iulia.gallo98@e-uvt.ro (I.G.); camelia.epuran@e-uvt.ro (C.E.); raul.stefan00@e-uvt.ro (R.Ș.-P.); alexandru.pahomi@e-uvt.ro (A.P.); mihaela.budiul@e-uvt.ro (M.M.B.); 2 Doctoral School of Exact Sciences and Natural Sciences, West University of Timișoara, Pestalozzi Street 16, 300115 Timisoara, Romania

**Keywords:** κ-carrageenan hydrogels, phenprocoumon, controlled drug release, HPLC, thermal analysis

## Abstract

Oral administration of narrow therapeutic index anticoagulants like phenprocoumon (PHP) necessitates careful control of the kinetic release of the drug to avoid dose dumping and severe haemorrhagic effects. This study was carried out to prepare and characterize novel PHP delivery systems based on κ-carrageenan hydrogels, exploring the importance of potassium ion (K^+^) stabilization in controlling the release process. FT-IR, TG/DTG, and in vitro release studies were employed in combination with a new validated RP-HPLC assay. FT-IR and thermal analysis results showed that PHP is physically encapsulated into the polysaccharide matrix, where there are no chemical incompatibilities between them. Furthermore, potassium ions increase the stability and heat resistance of the polymer network. However, when K^+^ was considered for modelling the kinetic release using the Korsmeyer–Peppas equation, it was observed that PHP is released from the K^+^ stabilized matrix in a relaxation dominated diffusion-controlled transport. Ionic cross-linking effectively reduces the initial burst effect, demonstrating that these matrices are promising vehicles for the sustained delivery of phenprocoumon.

## 1. Introduction

Polysaccharide-based hydrogels are of great interest in the pharmaceutical and biomedical fields because of their biocompatibility, biodegradability, and ability to form three-dimensional networks that can retain large amounts of water or incorporate active substances [1]. In addition, these features offer high potential for controlled drug release because the polymer network of the hydrogels regulates the diffusion and release kinetics of biomolecules [2]. Hydrogels also have properties such as high thermal stability, the ability to absorb large amounts of water or biological fluids, and mechanical and swelling properties that mimic biological tissues [3]. Due to these remarkable properties, the current literature highlights a growing interest in exploring their potential for wound healing [4], biosensors [5], advanced biomedical applications [6] and controlled release of the drug [7].

Among the polysaccharides used for hydrogel preparation, κ-carrageenan stands out for its superior properties, which are attributed to its chemical structure rich in functional groups and its ability to form stable gel networks. This versatile biopolymer enables the development of hydrogel systems that can incorporate and release bioactive compounds in a controlled manner, thereby enhancing therapeutic efficacy and reducing adverse effects [8]. Notably, κ-carrageenan is included in the United States Pharmacopeia 35-National Formulary 30 S1 (USP35-NF30 S1), the British Pharmacopoeia 2012 (BP2012), and the European Pharmacopoeia 7.0 (EP7.0) [9]. This inclusion highlights its significant potential as a reliable pharmaceutical excipient [9]. From a toxicological perspective, the high-molecular-weight form of native κ-carrageenan is classified as safe (GRAS—Generally Recognized As Safe) by the FDA and other international regulatory authorities [10]. κ-Carrageenan is highly biocompatible and exhibits low systemic absorption following oral intake. Therefore, the use of high-molecular-weight κ-carrageenan ensures a high safety level for oral formulations [11]. Building on its established regulatory profile and exceptional gel-forming capabilities, the recent literature highlights a wide variety of k-carrageenan-based formulations designed for targeted and sustained drug delivery, as summarized in Table 1.

Analysis of the existing literature (Table 1) shows that most approaches to designing κ-carrageenan hydrogel formulations involve combining κ-carrageenan with additional polymers such as sodium alginate or PVA or introducing complex structures such as MgZnO nanoparticles or biologically derived nanostructures. Although these hybrid systems offer a wide range of possibilities, they may present additional challenges associated with more complicated manufacturing processes, as well as the use of nanomaterials, which raises safety concerns due to potential nanoparticle toxicity [17]. The strategy proposed in this study utilizes a straightforward and safer method that exploits the intrinsic ionotropic gelation properties of κ-carrageenan. The addition of potassium ions in κ-carrageenan increases the cross-linking density and mechanical resistance of the polymer network. K^+^ has an optimal ionic radius that fits perfectly within the cavities of the κ-carrageenan double helices, effectively shielding the electrostatic repulsion between the negatively charged sulphate groups. By using only K^+^ ions to form ionic cross-links, the helices can aggregate tightly into a dense three-dimensional network with high stability [18].

Phenprocoumon is a widely used oral anticoagulant [19,20]. Structurally, it belongs to the 4-hydroxycoumarin class. Conventional delivery faces significant challenges, including pharmacokinetic variability and a notably long half-life. Therefore, rigorous therapeutic monitoring is required for patient safety [21]. Phenprocoumon is highly lipophilic, with poor aqueous solubility but extremely high membrane permeability, resulting in oral bioavailability approaching 100% and fitting the Biopharmaceutics Classification System (BCS) Class II profile [22]. Pharmacologically, phenprocoumon acts as an anticoagulant by serving as a vitamin K antagonist. It reversibly inhibits the vitamin K epoxide reductase enzyme (VKORC1), preventing the activation of vitamin K-dependent coagulation factors II, VII, IX, and X [23]. Although highly effective, phenprocoumon has several clinical drawbacks, including a narrow therapeutic index, a half-life of up to 6 days, and a slow onset of action [21]. Because it crosses the intestinal epithelial barrier so efficiently, conventional immediate-release formulations risk generating rapid spikes in maximum plasma concentrations, significantly increasing the likelihood of severe hemorrhagic complications [21]. Incorporating this lipophilic drug within a hydrophilic, K^+^-stabilized κ-carrageenan hydrogel network physically constrains its diffusion. This structural entrapment effectively modulates initial swelling and suppresses rapid burst absorption, transforming it into a safe, controlled, and sustained release profile tailored for oral anticoagulant therapy [24]. Detailed characterization of the hydrogel matrix is essential to understand the underlying mechanisms. FT-IR spectroscopy identifies specific functional groups and clarifies intermolecular interactions between the polymer network and the active ingredient [7]. Additionally, thermogravimetric analysis (TGA) determines thermal stability limits and tracks the degradation pathways of the hydrogel matrix [25]. Comprehensive preformulation compatibility screening using FT-IR and thermal analysis is essential for phenprocoumon delivery systems. Our recent studies have shown that common solid dosage excipients such as starch, lactose, and polyols remain chemically compatible with the drug, whereas certain lubricants like magnesium stearate can induce physicochemicalinteractions with phenprocoumon [26]. This emphasizes the necessity of creating customized macromolecular matrices to guarantee solid-state compatibility.

Drug release evaluation is a critical step in characterizing these systems. Analytical methods such as HPLC and UV-Vis spectrophotometry are used to quantify the active substance and analyze its release kinetics [12,27].

Chemical characterization and quantification of phenprocoumon within the final system were performed using a validated High-Performance Liquid Chromatography (HPLC) assay. The results are expressed as a mass assay, specifically denoting the drug concentration relative to the total matrix mass (mg/g of final formulation) and the absolute drug content per individual dosage form (mg of API per unit). This analytical approach was selected to accurately reflect the structural evolution of the formulation during manufacturing. Unlike colloidal or vesicular delivery systems (such as nanoparticles or liposomes) that require the determination of encapsulation efficiency (EE) to differentiate between free and carrier-bound drug, the system developed in this study is a monolithic matrix. Because all formulation components are fully integrated and remain within the system after the volatile solvents evaporate, EE is inherently inapplicable. Therefore, the HPLC mass assay provides the most scientifically rigorous metric to confirm composition accuracy, processing stability, and overall content uniformity [27].

This study aimed to develop and characterize κ-carrageenan hydrogels loaded with phenprocoumon. We evaluated their structural, thermal, and release profiles to establish an efficient platform for controlled drug delivery.

## 2. Results and Discussion

### 2.1. Structural Characterization by FT-IR Spectroscopy of Phenprocoumon-Based Hydrogels Stabilized and Non-Stabilized with Potassium Ions

Fourier transform infrared spectroscopy (FT-IR) analysis was used to identify the specific functional groups of the components used to obtain the hydrogel. This method also highlighted the interactions between constituents, the formation and architecture of the hydrogel network, specifically regarding the influence of phenprocoumon (PHP) incorporation and the impact of potassium ion-induced cross-linking.

**Figure 1:**
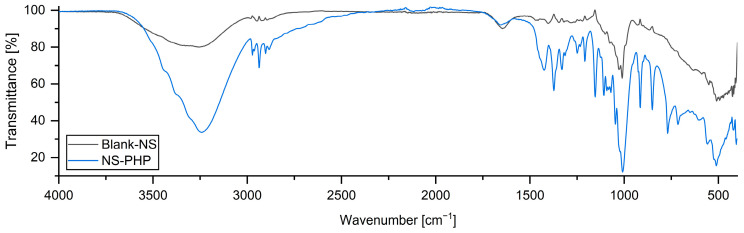
FT-IR spectra of non-stabilized κ-carrageenan hydrogels: comparison between the Blank-NS (drug-free control) and the NS-PHP (phenprocoumon-loaded formulation).

The FT-IR spectra of the blank hydrogel (Blank-NS) and the PHP-loaded hydrogel (NS-PHP5) are presented in Figure 1. The spectrum of the blank hydrogel exhibits a broad absorption band in the 3250–3330 cm^−1^ region, attributed to the –OH stretching vibrations of the hydroxyl groups within the polysaccharide structure. In the fingerprint region (1100–1000 cm^−1^), the characteristic bands of the κ-carrageenan backbone are observed at 1076, 1028, and 1010 cm^−1^, corresponding to the C–O and C–O–C stretching vibrations of the glycosidic linkages [28]. Additionally, the region between 500 and 420 cm^−1^ displays bands associated with the skeletal deformation of the polysaccharide chain and the sulphate groups.

In the spectrum of the PHP-loaded formulation (NS-PHP5), the characteristic bands of the κ-carrageenan matrix remain preserved, indicating that the polymer chain structure remains intact throughout the incorporation process. Crucially, new absorption bands emerge in the 1450–1200 cm^−1^ region, which are attributed to the C–H bending vibrations and C=C stretching vibrations. The aromatic character of PHP is evidenced by the band at 1425 cm^−1^, which corresponds to the skeletal C=C stretching vibrations of the aromatic ring. This peak is particularly useful for identifying the drug’s presence, as it does not overlap with the polysaccharide skeletal vibrations. The band at 1249 cm^−1^ is assigned to the asymmetric C–O–C stretching vibration of the coumarin moiety, while the signal at 1209 cm^−1^ is associated with the in-plane C–H bending vibrations characteristic of the aromatic substitution pattern of PHP. In the 900–700 cm^−1^ range, several diagnostically valuable bands are observed: the band at 769 cm^−1^ and the shoulder at 715 cm^−1^ are attributed to the out-of-plane C–H bending vibrations of the aromatic ring, providing another evidence of the PHP molecule’s structural integrity within the hydrogel. The presence of these specific peaks in the hydrogel formulations, absent in the blank samples, confirms the successful incorporation of phenprocoumon into the hydrogel network without chemical modification. Furthermore, a shift and broadening of the –OH stretching band (observed at approx. 3242 cm^−1^) suggest the existence of intermolecular hydrogen bonding between the polymeric matrix and the active substance. These observations confirm that the incorporation of phenprocoumon occurs through physical intermolecular interactions rather than the formation of new covalent bonds, preserving the inherent properties of the κ-carrageenan network [29,30]. The polyols utilized in our hydrogel core, namely mannitol and xylitol, are entirely inert and chemically compatible with phenprocoumon, showing no spectrum changes or chemical interactions even after prolonged storage, as reported in previous studies [26].

The absence of significant shifts in the characteristic polysaccharide absorption bands suggests that the κ-carrageenan polymer backbone remains structurally intact following drug incorporation. These findings indicate that phenprocoumon is embedded within the hydrogel matrix primarily through non-covalent physical intermolecular interactions, with no evidence of new covalent bond formation between the drug and the polymer network. This physical entrapment is consistent with previous reports on κ-carrageenan-based drug delivery systems, where the structural integrity of the gel matrix is preserved during the encapsulation process [24,31].

**Figure 2:**
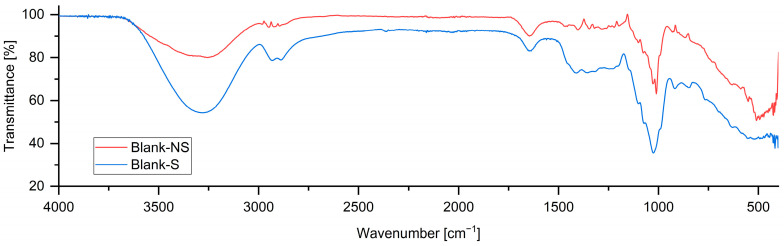
Comparative FT-IR spectra of κ-carrageenan-based hydrogel matrices: non-stabilized (Blank-NS) vs. K^+^-stabilized (Blank-S) formulations.

A comparative analysis of the FT-IR spectra for κ-carrageenan-based hydrogels without PHP, including both non-stabilized and K^+^-stabilized formulations (Figure 2), reveals the characteristic bands of the polysaccharide backbone. The preservation of these spectral features in both systems confirms that the underlying polymer chain structure remains intact after the stabilization treatment. Also, we can see the broad band in the region 3200–3400 cm^−1^, attributed to the –OH stretching vibrations, which becomes slightly more intense and broader in the presence of potassium ions. In addition, in the range of 1100–1000 cm^−1^, corresponding to the C–O and C–O–C stretching vibrations, the bands are better defined for the stabilized system, indicating a more compact organization of the polymer network. The changes observed in the region of 1200–900 cm^−1^ can be associated with ionic interactions between potassium ions and sulphate groups of carrageenan, contributing to the organization of the polymer chains in a more compact and stable network, specific to carrageenan-based gelled systems [29,32]. However, the lack of new bands or significant shifts in their positions suggests that the chemical structure of the polymer is not affected, and stabilization occurs mainly through intermolecular physical and ionic interactions, in agreement with the literature [7,24].

**Figure 3:**
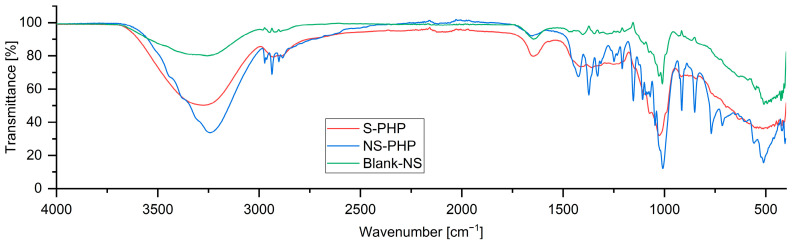
FT-IR spectra of κ-carrageenan-based hydrogels: Blank NS (non-stabilized control hydrogel), NS-PHP (phenprocoumon-loaded hydrogel, non-stabilized) and S-PHP (phenprocoumon-loaded hydrogel, stabilized with KCl).

The FT-IR spectra of Blank NS, NS-PHP, and S-PHP samples (Figure 3) display similar profiles, with no new bands appearing after loading with phenprocoumon and stabilization with K^+^ ions, indicating preservation of the chemical structure of the system components. However, compared to the NS-PHP sample, the spectrum of the S-PHP sample shows changes in the intensity and shape of the bands in the 1250–1000 cm^−1^ region, as well as a slight shift in the band from approximately 1425 cm^−1^ to 1418 cm^−1^. These variations, observed in the characteristic region of sulphate groups and glycosidic bonds of κ-carrageenan, suggest changes in the molecular environment of the polymer and are consistent with ionic interactions between potassium ions and sulphate groups. This behaviour aligns with the gelation mechanism reported for κ-carrageenan, in which potassium ions promote the conformational transition of the polymer chains to helical structures and their aggregation into a stable three-dimensional network [18].

### 2.2. Thermal Gravimetric Analysis

**Figure 4:**
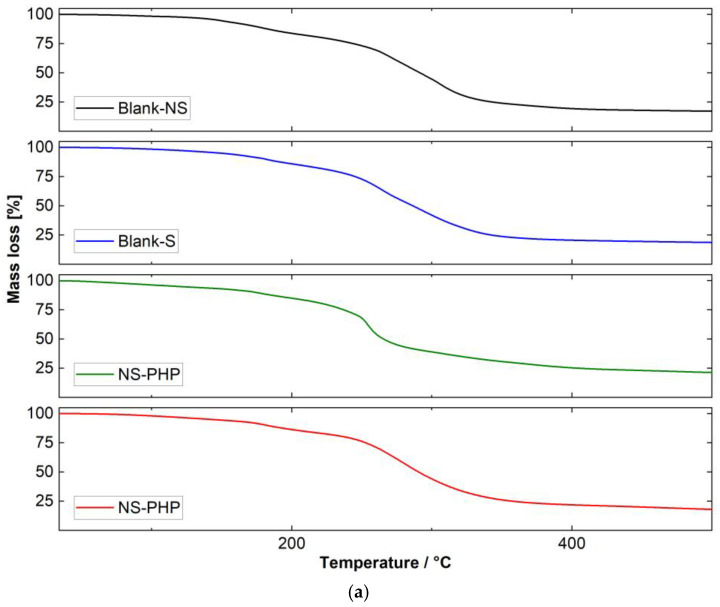

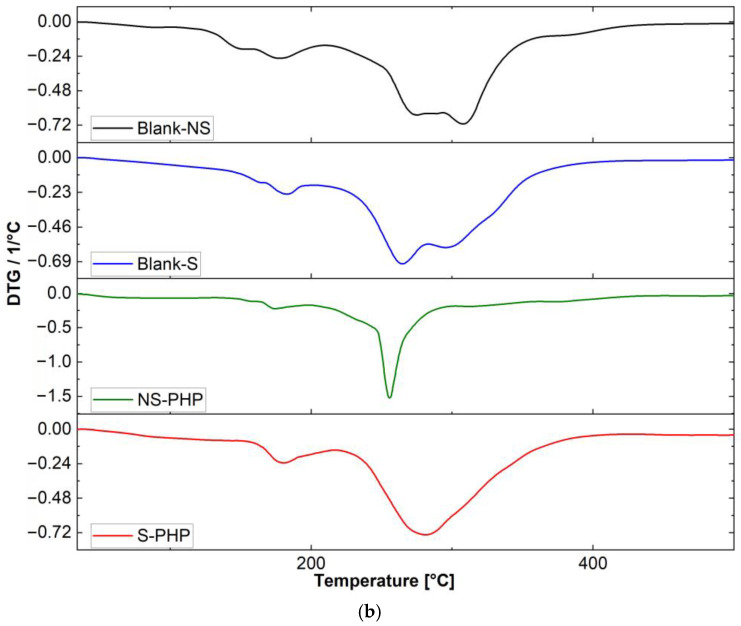
Thermoanalytical (**a**) TG and (**b**) DTG curves of κ-carrageenan-based hydrogels containing phenprocoumon, stabilized and non-stabilized with potassium ions.

The thermal behaviour of κ-carrageenan-based hydrogels containing the active substance (PHP) was investigated using TG/DTG analysis (Figure 4a,b) to evaluate thermal stability and degradation stages. The main thermoanalytical processes, with the corresponding mass losses, are presented in Table 2.

Analysis of the results obtained by FT-IR spectroscopy and thermogravimetric analysis (TG/DTG) indicates that the incorporation of phenprocoumon has a limited impact on the structure of the hydrogel network, without significantly altering the material’s thermal degradation behaviour. Although some differences were observed in band intensity, mass loss distribution, and the temperatures associated with individual degradation steps, the overall degradation profile of the hydrogel matrix remained comparable across the investigated formulations. Additionally, hydrogels stabilized with potassium ions showed slight improvements in structural organization and thermal resistance, indicating stronger intermolecular and ionic interactions within the network [7,24,33].

These results confirm the physicochemical compatibility of phenprocoumon with the κ-carrageenan-based hydrogel matrix and may suggest its utility in controlled-release applications of the active substances.

### 2.3. HPLC Method Validation and Drug Content Assay

Most reported HPLC methods utilize a reverse-phase chromatographic separation, typically employing C18 columns with a binary mobile phase system composed of aqueous buffers (such as phosphate, acetate or formate systems) combined with organic modifiers, primarily methanol or acetonitrile. Based on the available literature, a new and simple RP-HPLC method was developed and validated according to ICH-Q2 guidelines [34,35].

#### 2.3.1. Chromatographic Conditions

The aqueous mobile phase (A) consisted of a 50 mM solution of ammonium formate in ultrapure water, acidified with 0.4% (*v*/*v*) Formic acid. The organic mobile phase (B) consisted of an 80:20 (*v*/*v*) mixture of gradient-grade acetonitrile and ultrapure water. The column selected for this method was an Agilent Technologies Zorbax Eclipse XDB C18 150 mm × 4.6 mm, 5 µm particle size column (USP L1). Column conditioning was performed by increasing the aqueous mobile phase (A) concentration at a rate of 5% (*v*/*v*)/min until reaching the initial method conditions (60% A/40% B), followed by an equilibration period of 1 h prior to sample injection. The separation is performed by using a gradient programme (Table 3). An injection volume of 5 µL was used to ensure good repeatability and to minimize column loading. The column temperature was maintained at 40 °C throughout the analysis to eliminate the impact of ambient laboratory temperature fluctuations, thereby ensuring highly reproducible retention times and stable baselines. Furthermore, the elevated temperature reduces mobile phase viscosity, which lowers system backpressure and enhances mass transfer. This optimization ultimately yielded sharper peak shapes, improved chromatographic resolution, and minimized the overall analysis time for phenprocoumon.

**Figure 5:**
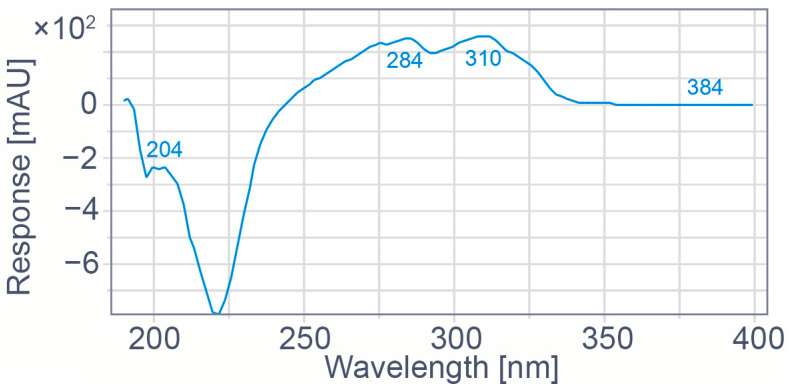
DAD absorption spectrum of the phenprocoumon peak corrected for baseline contribution by subtracting the baseline slope determined at the start and end of the elution window from the apex spectrum.

**Figure 6:**
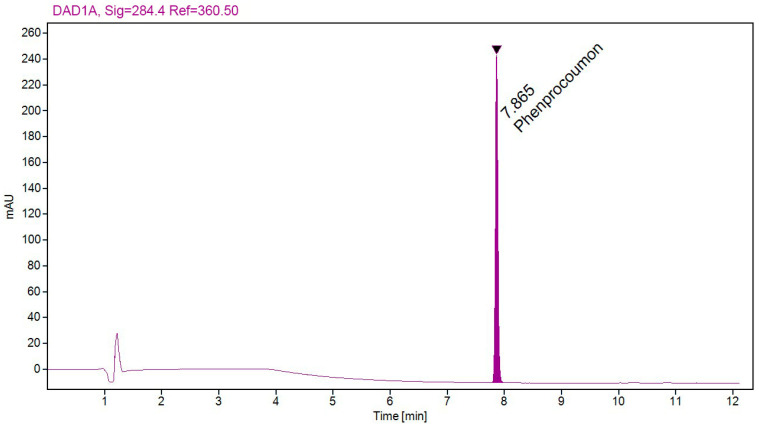
Reference chromatogram recorded on phenprocoumon SST.

Chromatograms were recorded at 284 nm (4 nm bandwidth), corresponding to one of the absorption maxima of phenprocoumon (Figure 5—spectrum and Figure 6—chromatogram). A reference wavelength of 360 nm (50 nm bandwidth) was used to correct baseline fluctuations arising from mobile phase composition changes and detector drift, thereby improving signal stability and integration precision.

#### 2.3.2. Method Validation

- System suitability

The system suitability results (Table 4) demonstrated that the chromatographic system was performing adequately for the phenprocoumon analysis. The %RSD for the peak area obtained from six replicate injections was 1.048%, which is within the acceptable limit of not more than 2.0%, indicating good repeatability. The retention time showed excellent consistency with a %RSD of 0.049%. In addition, the tailing factor values ranged from 1.056 to 1.109, confirming acceptable peak symmetry. The capacity factor (k’) was consistently approximately 6.87, indicating appropriate retention of the analyte. Theoretical plate counts were greater than 155,000 for all injections, demonstrating high column efficiency. Therefore, all system suitability parameters met the established acceptance criteria, confirming that the analytical system was suitable for the analysis.

- Linearity

**Figure 7:**
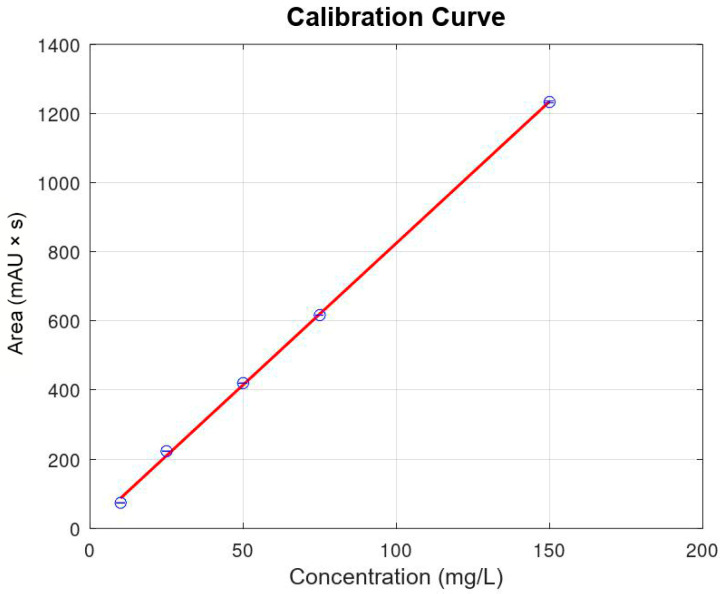
Linear regression plot of concentration versus peak area for the HPLC assay of phenprocoumon.

The linearity of the HPLC assay was evaluated by analyzing calibration standards at five concentration levels ranging from 10 to 150 µg/mL (Table 5). A strong linear relationship was observed (Figure 7) between analyte concentration and chromatographic peak area, as demonstrated by the determination coefficient (r) of 0.9995. These results confirm that the method exhibits excellent linearity within the studied concentration interval and is suitable for reliable quantitative analysis.

- Intermediate precision

The intermediate precision of the developed HPLC method for phenprocoumon was evaluated by determining the intraday and interday precision at three concentration levels (120, 150, and 180 µg/mL). Precision was expressed as percentage relative standard deviation (%RSD, *n* = 3). The intraday precision values ranged from 0.413% to 0.733%, while the interday precision values ranged from 0.287% to 0.641% (Table 6). The low %RSD values obtained at all concentration levels indicate that the proposed HPLC method demonstrates excellent repeatability and intermediate precision, confirming the reliability and reproducibility of the analytical procedure for the quantitative determination of phenprocoumon.

- Accuracy

The accuracy of the developed HPLC method was evaluated through recovery studies performed at three concentration levels (80%, 100%, and 120%) of analyte expected concentration. The spiked amounts ranged from 0.833 to 1.250 mg. The corresponding measured values ranged from 0.835 to 1.274 mg. The percentage recovery values (calculated using Equation (1)) were found to be between 98.94% and 101.94%, indicating excellent agreement between the theoretical and experimental results. All recovery values were well within the predefined acceptance criterion of ±2%, confirming the accuracy of the method. Furthermore, the %RSD values (0.36–0.72%) were low, demonstrating good repeatability and precision (Table 7).

(1) $$Recovery\left(\%\right)=\frac{{Measured}_{Amount}}{{Spiked}_{Amount}}\times 100$$

- Sensitivity

Method sensitivity was evaluated based on the signal-to-noise (S/N) ratio. The limit of detection (LOD) for phenprocoumon was determined as the concentration corresponding to an S/N ratio of 3 and was found to be 0.041 µg/mL. The limit of quantification (LOQ) for this method was calculated to be 0.135 µg/mL.

(2) LOQ=3.3×LOD

- Specificity and selectivity

**Figure 8:**
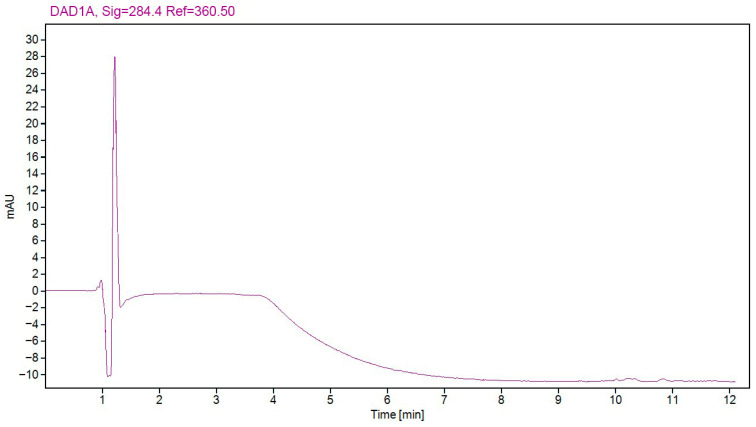
Methanol solvent blank chromatogram.

**Figure 9:**
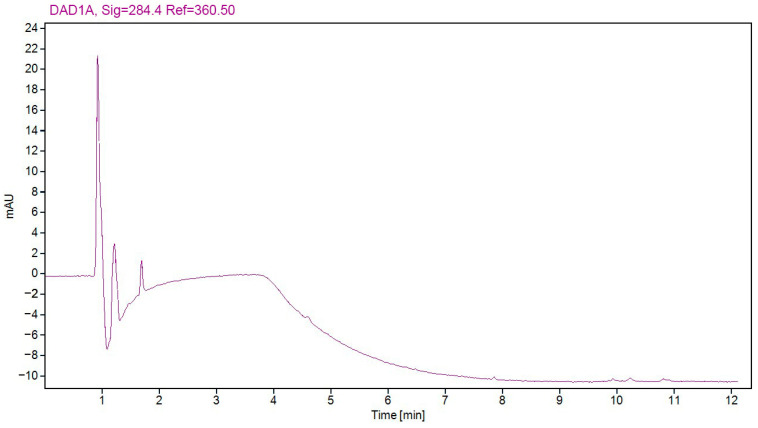
Matrix blank chromatogram of excipients (without API).

**Figure 10:**
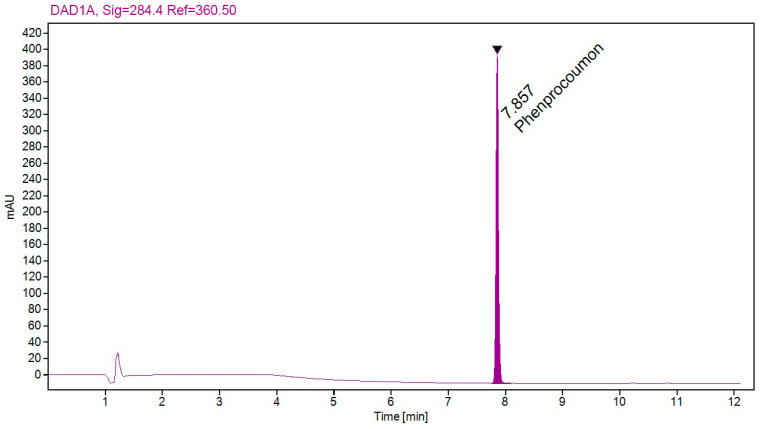
Phenprocoumon API standard chromatogram.

**Figure 11:**
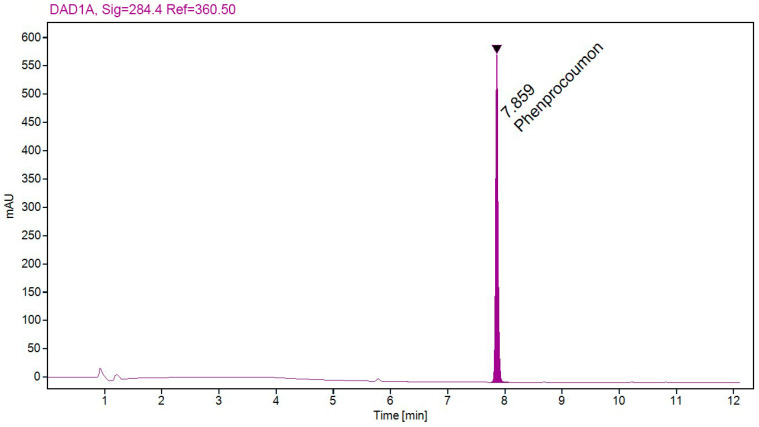
Excipients spiked with phenprocoumon chromatogram.

Specificity and selectivity of the proposed HPLC method were evaluated by analyzing the methanol solvent (Figure 8), a matrix blank containing excipients (Figure 9), a standard solution of phenprocoumon (Figure 10), and excipients spiked with the active pharmaceutical ingredient (Figure 11). The chromatographic results showed no interference from excipients at the retention time of phenprocoumon, demonstrating adequate specificity of the method. In addition, UV peak purity analysis using diode array detection (DAD) showed purity values close to 1000 for phenprocoumon peaks across all tested samples, indicating excellent spectral homogeneity. These results demonstrate that the method is selective and suitable for the accurate determination of phenprocoumon in pharmaceutical formulations.

- Method robustness

Robustness of the developed HPLC method was evaluated by deliberately varying the mobile phase flow rate by ±10% from the optimized condition. The effect of these changes on chromatographic performance was assessed using retention time, peak symmetry, and theoretical plate count (Table 8). Minor variations in retention time were observed as expected, however, no significant changes in assay results, peak shape, or system suitability parameters were detected.

#### 2.3.3. Sample Results

The assay results obtained (Table 9) for the phenprocoumon-loaded hydrogel formulations demonstrated excellent agreement with the target drug content values for both PHP3 and PHP5 samples. The PHP3 formulations showed assay values of 2.932 mg and 3.096 mg, corresponding closely to the theoretical target content of 3 mg per unit. Similarly, the PHP5 formulations yielded assay values of 4.914 mg and 5.143 mg, which are in good agreement with the expected 5 mg loading. All measured values were found to be within the predefined acceptance criterion of ±5% relative to the target content, confirming the accuracy and suitability of the developed HPLC method for quantitative analysis of phenprocoumon hydrogel formulations.

(3) Assay(mgg)=SampleArea−InterceptSlope×1000×SolventVolume×DilutionFactor

(4) Assay(mg)=Assay(mgg)1000×Samplemass (mg)

### 2.4. In Vitro Release Profile of Phenprocoumon from κ-Carrageenan Hydrogels

Drug release experiments were conducted using a dissolution apparatus at 37 °C and a stirring speed of 50 rpm. The release medium was 200 mL of phosphate buffer (pH = 7), simulating the environment of the small intestine. A calibration curve was established using pure phenprocoumon dissolved in the same buffer. The 310 nm wavelength was selected for quantification because it is the absorption maximum for PHP within the 250–800 nm range. This band was highly stable and unaffected by pH fluctuations. Additionally, the 310 nm region showed no spectral interference, as the hydrogel excipients did not absorb at this wavelength. To ensure reproducibility and instrument sensitivity, the calibration was performed in triplicate with independently prepared solutions. Also, the results obtained by UV-Vis were compared with those determined by HPLC to validate the analytical method. The UV-Vis absorption spectra for PHP solutions in the concentration range 7.8 × 10^−7^ M to 2 × 10^−4^ M, as well as the calibration curve, are provided in the Supplementary Materials in Figure S1 and Figure S2, respectively.

**Figure 12:**
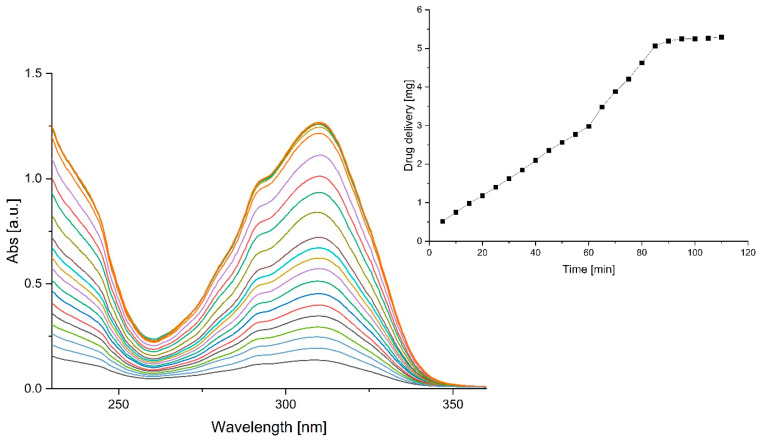
Superimposed UV-Vis absorption spectra showing the time-dependent increase in absorbance and the resulting cumulative drug release profile for the K-stabilized 450 mg hydrogel (~5 mg PHP loading), determined via periodic sampling at 310 nm.

**Figure 13:**
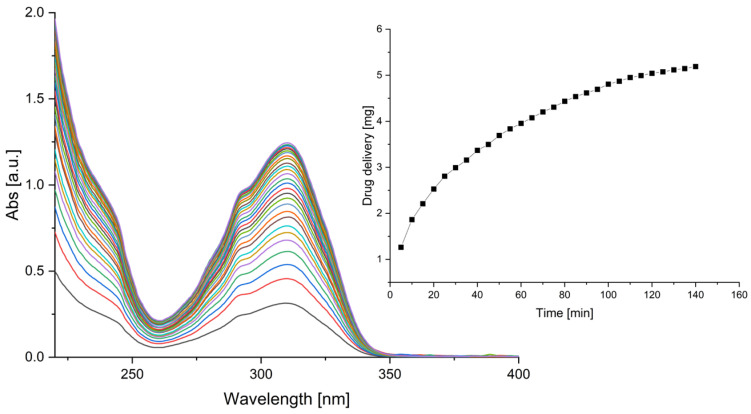
Superimposed UV-Vis absorption spectra and cumulative drug release profile for the non-stabilized 450 mg hydrogel (5 mg PHP loading), illustrating the rapid initial release (burst effect) determined via periodic sampling at 310 nm.

The release kinetics were evaluated for four distinct hydrogel formulations, comparing stabilized (with K^+^ ions) and non-stabilized κ-carrageenan matrices at two drug loading levels. Initial experiments involved periodic sampling and full UV-Vis spectral scans to monitor the release from the loaded hydrogels. s. For the K^+^-stabilized formulation, a hydrogel weighing 450 mg achieved a cumulative release of approximately 5.29 mg after 110 min (Figure 12). This profile demonstrates that the stabilized matrix ensures controlled and sustained diffusion of the active substance throughout the experiment. Similar release patterns were observed for the 3 mg PHP loading, confirming that the controlled-release behaviour remains consistent across different drug concentrations. In contrast, the non-stabilized hydrogel showed a more rapid initial release, reaching a similar plateau but with faster early-stage kinetics (Figure 13).

#### Drug Release Kinetics and Mechanism

To understand the release mechanism of PHP from the hydrogel matrices, the real-time dissolution data (up to 60% of total drug release) were fitted to the Korsmeyer-Peppas model (Equation (5)), where $\frac{M_{t}}{M_{\infty }}$ is the fraction of drug released at time t, k is the release rate constant, and n is the diffusional exponent indicating the transport mechanism [36].

(5) $$\frac{M_{t}}{M_{\infty }}=k\cdot t^{n},$$

The calculated exponent n confirmed that ionic cross-linking significantly alters the release behaviour. The K-stabilized hydrogels exhibited high n values compared to non-stabilized samples (n = 0.75 for the 5 mg loading and n = 0.94 for the 3 mg loading), with excellent correlation coefficients ($R^{2}\;>\;0.99$). These values indicate anomalous (non-Fickian) and Case II transport mechanisms, meaning drug release is largely governed by the gradual swelling and macromolecular relaxation of the κ-carrageenan network. The potassium ions effectively bind the double helices together, forcing the drug to release at a sustained rate controlled by polymer hydration. In contrast, the non-stabilized matrices yielded lower diffusional exponents (n = 0.71 and n = 0.62 for 5 mg and 3 mg loadings, respectively). This shift toward lower n values demonstrates reduced resistance to swelling. Without the stabilizing K^+^ bridges, rapid water penetration occurs, dilating the matrix pores immediately and allowing diffusion to play a more dominant role. This mathematically confirms the pronounced initial burst effect observed in the non-stabilized release profiles.

To rigorously validate the selection of the Korsmeyer–Peppas model and confirm the underlying transport mechanisms, the in vitro release data (up to 60% cumulative dissolution) were also fitted to four classical kinetic models: Zero-order, First-order, Higuchi, and Hixson–Crowell equations [36,37,38]. The resulting determination coefficients (R^2^) are summarized in Table S1 from Supplementary Materials.

**Figure 14:**
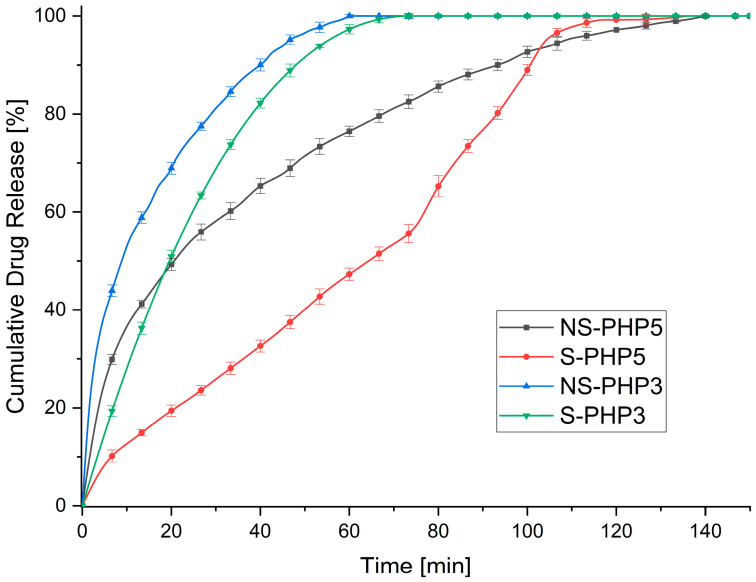
In vitro release profiles of phenprocoumon from non-stabilized (NS-PHP) and K^+^-stabilized (S-PHP) κ-carrageenan-based hydrogel matrices at drug loadings of 3 mg and 5 mg PHP in simulated intestinal fluid. Data are expressed as mean ± SD (*n* = 3 independent experiments).

The release behaviour of the NS-PHP and S-PHP hydrogels in simulated intestinal fluid is shown in Figure 14. All formulations displayed release profiles marked by a progressive increase in the cumulative amount of phenprocoumon, eventually reaching a sustained plateau. This behaviour confirms the hydrogel network’s ability to provide controlled drug delivery and demonstrates the stability of the investigated systems under intestinal conditions. Although the overall release profiles of the NS-PHP and S-PHP samples are comparable in terms of total drug delivered, distinct differences appear during the early stages of the dissolution process. The K^+^-stabilized hydrogels show a more controlled initial release phase compared to the non-stabilized samples, effectively reducing the initial rapid release. These results indicate that K^+^ stabilization specifically modifies the early-stage release kinetics by reinforcing the polymer network, without compromising the system’s overall capacity to achieve complete and sustained delivery of phenprocoumon.

The pronounced delay in release from the S-PHP5 formulation, compared with S-PHP3, is strongly influenced by the microenvironmental hydrophobicity of the matrix. Because phenprocoumon is highly lipophilic, increasing the drug loading from 3 mg to 5 mg within the same hydrogel mass inherently increases the overall lipophilicity of the polymeric core. This heightened hydrophobicity acts synergistically with K^+^ stabilization to actively repel water penetration, thereby slowing the hydration rate and macromolecular relaxation of the network.

It is noteworthy that, to simulate the small intestine, release behaviour was assessed only at pH 7.0. Our lab’s initial experiments in an acidic medium (pH 1.2) showed that the active ingredient was poorly solubilized. Phenprocoumon, a weakly acidic BCS Class II drug, exhibits minimal dissolution in the stomach because it remains largely unionized in highly acidic conditions. As a result, the primary site for its dissolution and subsequent absorption is at neutral pH. Future research will use pH-gradient dissolution testing to fully map the biorelevant release profile, but the current in vitro model is limited by the absence of a sequential gastrointestinal transit simulation (i.e., continuous exposure to gastric pH before intestinal pH).

## 3. Materials and Methods

### 3.1. Chemicals and Reagents

Phenprocoumon was used as the active pharmaceutical ingredient, produced by Farmexim, Romania. The excipients used in this study were: κ-carrageenan (Acros Organics, Geel, Belgium), mannitol was supplied by Merck, Germany, glucose (Lach-Ner S.R.O., Neratovice, Czech Republic), citric acid (Merck KGaA, Darmstadt, Germany), 1,3-propandiol (Merck, Darmstadt, Germany), ethyl alcohol (ChimReactiv S.R.L., București, Romania).

### 3.2. Hydrogel Synthesis

Hydrogels were prepared by thermal gelation using κ-carrageenan as the primary polymer matrix by adapting the protocol mentioned by Kim D. et al. [39]. Two distinct formulations were developed to achieve final therapeutic loadings of 3 mg and 5 mg of phenprocoumon (PHP) per 450 mg hydrogel unit. For the 3 mg PHP formulation, a solid blend of 0.60 g k-carrageenan, 15.04 g glucose, 4.14 g xylitol, and 1.50 g mannitol was thoroughly premixed to prevent polymer agglomeration. This mixture was gradually dispersed into 47.5 g of double-distilled water preheated to 90–95 °C. The system was maintained under continuous mechanical stirring (700–1000 rpm) for 20 min until complete polymer dissolution. Subsequently, 1.50 g of glycerine and 1.50 g of 1,3-propanediol were added as plasticizers to enhance matrix flexibility and water retention, after which the temperature was stabilized at 70–75 °C. For the 5 mg PHP formulation, the matrix components and water volumes were proportionally doubled (1.20 g κ-carrageenan, 30.09 g glucose, 8.28 g xylitol, 3.00 g mannitol, and 95.0 g water, along with 3.00 g of each plasticizer).

The active ingredient was incorporated using a co-solvent technique. Specifically, 0.125 g of PHP (for the 3 mg formulation) or 0.50 g of PHP (for the 5 mg formulation) was dissolved in 7.5 g and 9.67 g of 96% ethanol, respectively. The ethanolic drug solution was added dropwise to the hot polymer base under gentle agitation to ensure homogeneous drug distribution and prevent aeration. Finally, the system’s pH was adjusted with a concentrated aqueous solution of citric acid monohydrate to ensure uniformity.

The hot, homogeneous mixture was cast into silicone moulds at 65 °C, then allowed to cool and partially dehydrate overnight at room temperature. During this curing phase, the ethanol and a substantial portion of the water evaporated, effectively concentrating the polymeric matrix. The samples were then stored at 4 °C for 12 h to complete the gelation process. As a result of this controlled solvent evaporation, the final cross-linked matrix achieved the precise target loadings of 3 mg and 5 mg of PHP per 450 mg of the finished hydrogel unit.

#### Optimizing the Structural Stability of the Hydrogel

To further enhance the structural integrity of the hydrogels, a parallel set of samples underwent post-preparation ionic cross-linking treatment. After a 48 h curing period, the hydrogels were immersed in a 2 M KCl solution for 1 min. This post-treatment was specifically applied to promote the formation of ionic bridges between the κ-carrageenan chains. The introduction of potassium ions increases the cross-linking density and mechanical resistance of the polymer network, leveraging the well-documented cation-specific aggregation of κ-carrageenan helices [40].

Six distinct hydrogel formulations were developed and analyzed to isolate the individual effects of drug loading and ionic stabilization on the structural and release properties of the matrices. These formulations, categorized by drug content (0 mg, 3 mg, or 5 mg PHP per 450 mg hydrogel unit) and stabilization status, are summarized in Table 10.

### 3.3. Apparatus

#### 3.3.1. FT-IR Spectroscopy

FT-IR analyses were conducted using a Shimadzu FTIR IRTracer-100 spectrometer (Shimadzu Corporation, Tokyo, Japan) equipped with a QATR-10 single-reflection ATR accessory with a diamond crystal, covering the spectral range of 4000–280 cm^−1^. Data were collected after 20 scans at a resolution of 4 cm^−1^.

#### 3.3.2. Thermogravimetric Analysis

Thermal analysis of the samples (~5–10 mg) was performed using a Mettler Toledo TGA/DSC3+ thermogravimetric analyser (Mettler-Toledo LLC, Columbus, OH, USA) over a temperature range of 25–500 °C, with a heating rate of 10 °C·min^−1^, under an air atmosphere at a flow rate of 50 mL/min, in open aluminum crucibles. The equipment was operated in accordance with the manufacturer’s specifications and standard calibration procedures.

#### 3.3.3. HPLC

Chromatographic analysis was carried out using an Agilent Technologies 1100 Series system (Agilent, Waldbronn, Germany) comprising a G1379A vacuum degasser, G1312A Binary Pump, G1311A autosampler, G1316A column thermostat, and G1315A diode array detector (DAD). Data acquisition and processing were carried out using Agilent OpenLab CDS (v2.8). Ultrapure water was prepared using a Merck Milli-Q purification system and it was changed daily during this study. Gradient-grade acetonitrile and methanol was supplied by Merck, Darmstadt, Germany. Ammonium formate was purchased from Sigma-Aldrich, Burlington, MA, USA, while formic acid was sourced from Honeywell Riedel-de Haën, Seelze, Germany.

- Mobile phase preparation

*Mobile phase A*—3.15 g of ammonium formate was weighed and transferred into a clean 1 L beaker, followed by the addition of 800 mL ultrapure water. The solution was stirred using a magnetic hotplate stirrer at 800 rpm for 10 min until complete dissolution. The resulting solution was quantitatively transferred into a 1 L volumetric flask, rinsing the beaker three times with 20 mL ultrapure water. Subsequently, 4 mL of Formic acid was added, and the solution was brought to volume with ultrapure water. The prepared mobile phase was filtered through a 0.45 μm nylon membrane filter suitable for HPLC analysis, transferred into an HPLC solvent bottle, and degassed in an ultrasonic bath at room temperature for 15 min prior to use.

*Mobile phase B*—A mixture of acetonitrile and ultrapure water (80:20, *v*/*v*) was prepared by transferring 800 mL acetonitrile and 200 mL ultrapure water into a clean 1 L HPLC solvent bottle. The mixture was thoroughly homogenized and subsequently degassed in an ultrasonic bath at room temperature for 20 min prior to use.

- Preparation of standard solution

The standard solution was prepared by weighing 9.97 mg of phenprocoumon reference standard into a 10 mL volumetric flask using an analytical balance. The standard was dissolved in approximately 8 ± 0.02 mL of methanol by sonication at room temperature for 5 min, after which the solution was brought to volume with methanol.

- System suitability test solution (SST)

The system suitability test (SST) solution was prepared by weighing 9.92 mg of phenprocoumon reference standard into a 10 mL volumetric flask using an analytical balance. The compound was dissolved in approximately 8 mL of methanol under sonication at room temperature for 5 min, after which methanol was added up to the mark. This stock solution was subsequently diluted 1:10 (*v*/*v*) by transferring 1 ± 0.02 mL into a 10 mL volumetric flask and making up to volume with methanol, yielding the working SST used for analysis.

- Preparation of sample solution

One hydrogel unit was accurately weighed and sectioned into small pieces using a clean scalpel. The resulting fragments were quantitatively transferred into a 10 mL volumetric flask, followed by the addition of 7 ± 0.02 mL of methanol as the extraction solvent. Extraction was performed by sonication at 40 °C for 45 min. After sonication, the solution was allowed to cool to room temperature and then transferred to a clean beaker. The solution was filtered through a syringe fitted with a 0.45 μm PTFE membrane filter, discarding the first few drops of filtrate. The filtrate was then directly used for further dilution steps as required (PHP3 and PHP5 preparations) prior to HPLC analysis. All solutions were prepared freshly and analyzed promptly to ensure consistency and prevent potential degradation.

- PHP3 sample solution preparation

The PHP3 sample solution (dilution factor 3) was prepared by transferring 5.00 mL ± 0.02 mL the filtered extract into a 15 mL volumetric flask. The solution was diluted to volume with methanol and mixed thoroughly to ensure homogeneity. The resulting solution was used directly for HPLC analysis without further treatment.

- PHP5 sample solution preparation

The PHP5 sample solution (dilution factor 10) was prepared by accurately pipetting 1.00 ± 0.02 mL of the filtered extract into a 10 mL volumetric flask. The solution was then made up to volume with methanol and mixed well prior to analysis. The prepared solution was injected directly into the HPLC system.

#### 3.3.4. UV-Vis Spectroscopy

The UV–Vis absorption measurements were performed using a Cary 60 UV–Vis spectrophotometer (Agilent Technologies, Santa Clara, CA, USA), employing 1 cm path length quartz cuvettes. The instrument was used for the quantitative determination of the analyzed compounds based on absorbance measurements at specific wavelengths.

Dissolution studies were carried out using a Agilent Technologies 708-DS Dissolution Apparatus (Agilent Technologies, Santa Clara, CA, USA). The system was employed to evaluate the release profile of the active compound under controlled experimental conditions, including temperature and stirring rate, according to the dissolution testing protocol. Samples withdrawn at predetermined time intervals were subsequently analyzed by UV–Vis spectrophotometry.

### 3.4. Statistical Analysis

All quantitative experiments, including HPLC assays and in vitro drug release studies, were performed in triplicate using independent batches. Data are expressed as mean values ± standard deviation (SD). Linear regression analysis for method validation was performed using Microsoft Excel 2016 and OriginPro 2021 (Version 9.8) software.

## 4. Conclusions

The current study has successfully shown that it is possible to formulate and comprehensively analyze the biopharmaceutical properties of κ-carrageenan hydrogels as an innovative drug delivery system for phenprocoumon (PHP). By means of structural, thermal, and chromatographic analysis, we demonstrated the importance of K^+^-induced ionic cross-linking in optimizing the delivery system’s properties. FT-IR and TG/DTG analyses showed that the API was physically incorporated into the polymer matrix via non-covalent interactions. Moreover, the cross-linking of polymer chains resulted in increased structural compactness and improved thermal resistance of the hydrogel systems. Drug loading and content uniformity were determined by a newly validated, highly sensitive RP-HPLC technique. Most importantly, mathematical modelling using the Korsmeyer–Peppas equation allowed us to demonstrate that K^+^ stabilization modulates the release kinetics by shifting the diffusional exponents (*n*) toward higher values. This transition indicates that K^+^ ions effectively reinforce the polymer network, forcing the release mechanism to become more dependent on macromolecular relaxation rather than simple diffusion, thereby effectively mitigating the initial burst effect risk associated with BCS class II drugs. In conclusion, while K^+^-stabilized κ-carrageenan hydrogels successfully mitigate the initial burst effect and exhibit highly predictable in vitro release kinetics, they represent a promising foundational platform that warrants comprehensive in vivo pharmacokinetic evaluations to fully confirm their clinical safety and therapeutic efficacy.

## Figures and Tables

**Table 1:** Recent examples of κ-carrageenan-based hydrogel formulations developed for the controlled release of various pharmacological agents.

Active Pharmaceutical Ingredient (API)	Hydrogel Composition/Cross-Linking Strategy	Delivery Route/Target	Main Outcome/Kinetics	Reference
Methotrexate	κ-carrageenan/Sodium alginate	Oral/ Colon-targeted	pH-responsive sustained release	[7]
Bromelain	κ-carrageenan/Nanofillers	Oral/Systemic	Diffusion-controlled release	[12]
Curcumin	κ-carrageenan/Alginate/Poloxamer	Oral/Intestinal	Enhanced bioavailability and controlled release	[13]
Catechin	κ-carrageenan/PVA/MgZnO NPs	Transdermal/Wound	Antimicrobial and sustained release	[14]
Celecoxib	κ-carrageenan	Site-specific/Anti-inflammatory	Triggered disintegration and accelerated release	[15]
Theophylline	κ-carrageenan functionalized with cinnamate groups (k-Crg-cinnamate (17–49%))	Oral	pH-responsive controlled drug release	[16]
Phenprocoumon	κ-carrageenan/K^+^ stabilization	Oral/Systemic	Suppression of burst effect/Sustained delivery	This work

**Table 2:** Thermogravimetric analysis (TG/DTG) parameters of κ-carrageenan-based hydrogels: characteristic temperatures and mass losses.

Sample	Process	TG	DTG_max_/°C	∆*m*/%
		*T*_onset_–*T*_final_/°C		
PHP	I	170–330	287, 293	97.48
Blank-NS	I	25–100	–	1.29
	II	100–210	148, 178	11.73
	III	210–420	266, 273, 304	52.47
Blank-S	I	25–100	–	1.35
	II	100–210	163, 175	6.63
	III	210–420	258, 266, 295	55.15
NS-PHP	I	25–100	–	3.25
	II	100–210	168	2.04
	III	210–410	255	44.92
S-PHP	I	25–100	–	2.32
	II	100–210	179	5.93
	III	210–410	185	53.68

**Table 3:** Method gradient program at flow 1.30 mL/min and post run time of 7 min.

Time (min)	%A	%B
0.00	60.00	40.00
2.00	60.00	40.00
8.00	0.00	100.00
12.00	0.00	100.00
12.10	60.00	40.00

**Table 4:** System suitability data.

Injection	Area of Target Peak (mAU × s)	Retention Time (min)	Tailing Factor	Capacity Factor (k’)	EP Plates (N)
1	781.406	7.873	1.084	6.87	155,602.33
2	774.652	7.865	1.076	6.87	156,958.53
3	774.852	7.865	1.065	6.87	156,116.61
4	793.559	7.864	1.109	6.86	157,146.72
5	775.287	7.864	1.056	6.87	155,981.07
6	770.812	7.862	1.107	6.86	157,497.43
Average	778.428	7.866	1.083	6.87	156,550.45
%RSD	1.048	0.049			

**Table 5:** Linearity data.

Concentration (µg/mL)	Area ± SD (mAU × s)	Calc. Concentration ± SD (µg/mL)	Slope	Intercept	Det. Coef.
10	73.878 ± 0.145	9.132 ± 0.018	8.1921	5.4589	0.9995
25	223.248 ± 0.117	27.004 ± 0.014			
50	420.201 ± 0.478	50.581 ± 0.058			
75	616.581 ± 0.483	74.087 ± 0.059			
150	1232.945 ± 2.727	147.876 ± 0.333			

**Table 6:** Intermediate precision data.

Analyte	Concentrations (µg/mL)	Intraday Precision 1st Day	Interday Precision 2nd Day
		%RSD (*n* = 3)	%RSD (*n* = 3)
Phenprocoumon	120	0.733	0.287
	150	0.553	0.641
	180	0.413	0.375

**Table 7:** Accuracy data.

Level %	Spiked Amount (mg)	Average Area (*n* = 3) (mAU × s)	Measured Amount (*n* = 3) (mg)	%RSD (*n* = 3)	Recovery % (*n* = 3)
80	0.833	982.862	0.835	0.47	100.23
100	1.042	1211.599	1.031	0.72	98.94
120	1.250	1496.642	1.274	0.36	101.94

**Table 8:** Robustness evaluation of the HPLC method.

Parameter	Variation	Symmetry	Retention Time (min)	EP Plates (N)	Tailing Factor
Flow	−10% mL/min	0.881	8.144	159,587.90	1.134
	+10% mL/min	0.899	7.513	148,638.05	1.065

**Table 9:** Sample results table.

Sample Number	Sample Name	Sample Mass (mg)	Average (*n* = 2) Area (mAU × s)	Assay (mg/g)	Assay (mg)
1	Blank-NS	693.40	N/A	N/A	N/A
2	Blank-S	842.17	N/A	N/A	N/A
3	NS-PHP3	689.23	1633.261	4.254	2.932
4	NS-PHP5	463.36	1300.315	10.605	4.914
5	S-PHP3	627.10	1767.369	4.937	3.096
6	S-PHP5	445.97	1306.160	11.532	5.143

**Table 10:** Experimental group designations based on drug loading and post-preparation ionic cross-linking.

Formulation Code	Loading (PHP)	Stabilization
Blank-NS	0 mg	Non-stabilized
Blank-S	0 mg	K^+^-stabilized
NS-PHP3	3 mg	Non-stabilized
NS-PHP5	5 mg	Non-stabilized
S-PHP3	3 mg	K^+^-stabilized
S-PHP5	5 mg	K^+^-stabilized

## Data Availability

The data presented in this study are available on request from the corresponding authors.

## Supplementary Materials

The following supporting information can be downloaded at: https://www.mdpi.com/article/10.3390/molecules31142540/s1, Figure S1: Overlapped UV-Vis absorption spectra of phenprocoumon (concentration range between 7.8 × 10^−7^ M and 2 × 10^−4^ M) in phosphate buffer medium (pH 7), and the calibration curve of phenprocoumon determined at λ = 310 nm; Figure S2: UV-Vis calibration curve of phenprocoumon determined at λ = 310 nm.; Table S1: Determination coefficients (R^2^) of the kinetic models evaluated for phenprocoumon release.

## Abbreviations

The following abbreviations are used in this manuscript:

PHP	Phenprocoumon
FT-IR	Fourier-Transform Infrared Spectroscopy
TG/DTG	Thermogravimetric/Derivative Thermogravimetric Analysis
HPLC	High-Performance Liquid Chromatography
LOD	Limit of Detection
LOQ	Limit of Quantification
