# Supplementary material for: Formulation and Analytical Characterization of Phenprocoumon-Loaded κ-Carrageenan Hydrogels for Controlled-Release Applications
Source: Molecules. 2026 Jul 22;31(14):2540. doi: 10.3390/molecules31142540 (PMC13415100; doi:10.3390/molecules31142540)
Supplement: Supplementary file 1 [file molecules-31-02540-s001.zip › molecules-4376557-supplementary.pdf]

Supplementary Materials

Formulation and Analytical Characterization of Phenprocoumon-Loaded κ-Carrageenan Hydrogels for Controlled Release Applications

Iulia Gallo [1,2], Camelia Epuran [1], Ion Fratilescu [1], Raul Ștefan-Pantiș [1,2], Alexandru Pahomi [1], Mihaela Maria Budiul [1], Titus Vlase [1,2]* and Gabriela Vlase [1,2]*

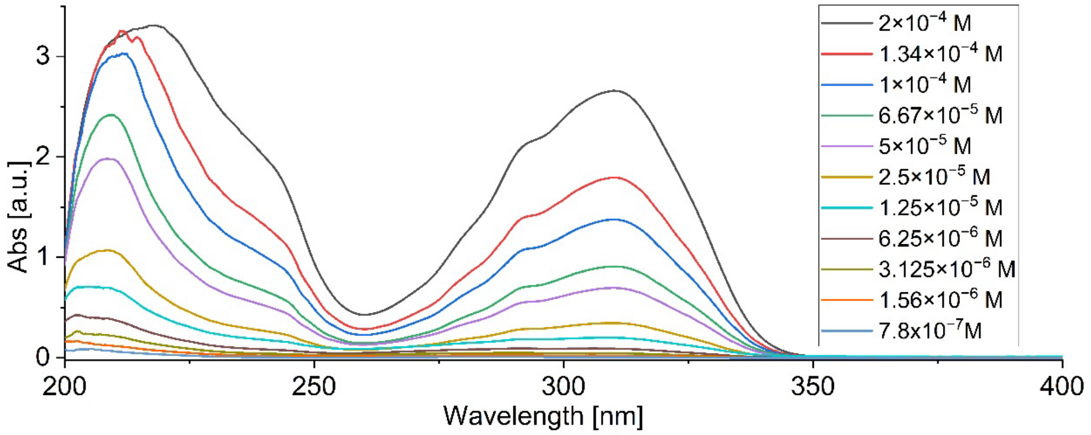

Figure S1. Overlapped UV-Vis absorption spectra of phenprocoumon (concentration range between 7.8×10^{-7} M and 2×10^{-4} M) in phosphate buffer medium (pH 7), and the calibration curve of phenprocoumon determined at $\lambda = 310$ nm.

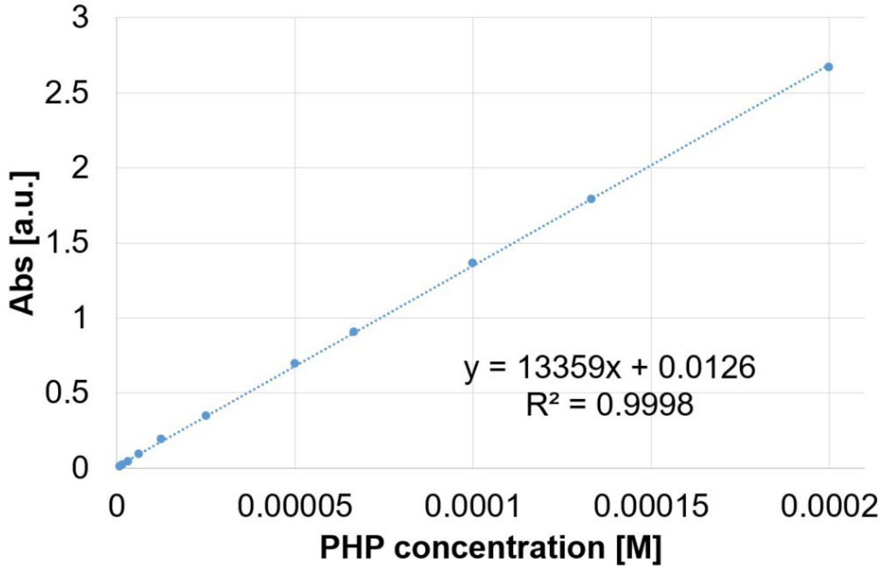

Figure S2. UV-Vis calibration curve of phenprocoumon determined at $\lambda = 310$ nm.

Table S1. Determination coefficients (R^2) of the kinetic models evaluated for phenprocoumon release.

Sample Code	Zero-Order (R^2)	First-Order(R^2)	Higuchi (R^2)	Hixson–Crowell (R^2)	Korsmeyer–Peppas (R^2)

NS-PHP3	0.9124	0.8845	0.9612	0.8992	0.9942
NS-PHP5	0.9255	0.8912	0.9587	0.9041	0.9918
S-PHP3	0.9418	0.8654	0.9403	0.8876	0.9972
S-PHP5	0.9532	0.8512	0.9312	0.8715	0.9964
